# Investigation of the Relationship between Compressive Strength and Hydrate Formation Behavior of Low-Temperature Cured Cement upon Addition of a Nitrite-Based Accelerator

**DOI:** 10.3390/ma12233936

**Published:** 2019-11-28

**Authors:** Jihoon Kim, Daiki Honda, Heesup Choi, Yukio Hama

**Affiliations:** 1 Department of Civil Engineering and Architecture, Muroran Institute of Technology, Hokkaido 0508585, Japan; bmjhun@mmm.muroran-it.ac.jp (J.K.); 19041073@mmm.muroran-it.ac.jp (D.H.); 2 Department of Civil and Environmental Engineering, Kitami Institute of Technology, Hokkaido 0908507, Japan; hs-choi@mail.kitami-it.ac.jp

**Keywords:** frost-resistant accelerator, calcium nitrite, nitrite based accelerator, hydrate formation, concrete strength, early compressive strength

## Abstract

When concrete is used for construction in cold-temperature regions, cold-resistant accelerators based on calcium nitrite (Ca(NO_2_)_2_) and calcium nitrate (Ca(NO_3_)_2_) are added to prevent early freezing damage. Although cold-resistant accelerators increase the early compressive strength and prevent early freezing damage by promoting cement hydration, the strength enhancement effect owing to the formation of such hydrates has not been evaluated quantitatively thus far. This study covers various types of analysis to understand the relationship between cement hydrate formation behavior and strength development upon the addition of varying amounts of nitrite-based accelerator. We find that the early compressive strength is enhanced by the addition of nitrite-based accelerator via the promotion of the relative production of monosulfate and C-S-H in the early age. However, the development of compressive strength decreases with an increase in the curing age. Furthermore, we find that the promotion of hydration reactions at an early age with the addition of nitrite-based accelerator can affect the formation ratio of each hydrate at a late age. We believe our findings can significantly contribute to developments in concrete application and allied fields.

## 1. Introduction

With regard to the use of concrete for construction in cold-weather countries, it is noteworthy that early frost damage caused by the freezing of water in concrete during the early stages may cause serious performance decline in quality of concrete [1,2]. Thus, it becomes necessary to control the temperature by heating the concrete for the period over which it reaches the “required early strength” to prevent initial frost damage [3]. However, in severely low-temperature environments or under conditions wherein it is difficult to set up a heater or a temporary tent owing to factors such as steep working slopes and narrow workplaces, a simple sheet curing method involving the addition of a frost-resistant accelerator is adopted [4,5,6,7].

Frost-resistant accelerators are admixtures that prevent early frost damage by promoting the hydration reaction of cement. They serve to lower the freezing temperature of water in the concrete and further accelerate concrete hardening, thereby increasing the early compressive strength [8]. In particular, nitrite–nitrate-based curing accelerators such as calcium nitrite (Ca(NO_2_)_2_) and calcium nitrate (Ca(NO_3_)_2_) are widely used as the main components of frost-resistant accelerators [9]. Nitrite–nitrate-based frost-resistant accelerators promote the hydration of tricalcium aluminate (C_3_A) and increase the amount of ettringite (AFt; 3CaO·Al_2_O_3_·3CaSO_4_·32H_2_O) and monosulfate (AFm; 3CaO·Al_2_O_3_·CaSO_4_·12H_2_O). Furthermore, it is known that hydrates such as nitrite hydrates (nitrite-AFm; 3CaO·Al_2_O_3_·Ca(NO_2_)_2_·xH_2_O) and nitrate hydrates (nitrate-AFm; 3CaO·Al_2_O_3_·Ca(NO_3_)_2_·xH_2_O) are formed through reactions between C_3_A and the NO_2_^−^ and NO_3_^−^ ions of the nitrite–nitrate-based frost-resistant accelerators [7,10,11]. Each hydrate that is generated enhances the early compressive strength and reduces early frost damage [1,9].

In regard to strength enhancement, Akama studied the development of a high-performance frost-resistant accelerator based on a large amount of calcium nitrite, whose addition can ensure sufficient hardening promotion and workability even at severely low temperatures [1]. They reported that such an accelerator is effective in early strength development relative to existing cold-resistance promoters. However, in other studies, it has been reported that the addition of a large amount of calcium nitrite is effective in improving the early strength, but the strength is lowered in the later ages [1,2,8,9]. There have been many similar studies on the effects of nitrite–nitrate-based accelerators on early strength development in low-temperature environments; however, in all these studies, the strength enhancement effect owing to the generation of such hydrates has not been evaluated quantitatively. Thus, it becomes necessary to gain an understanding of the effects of early-generated hydrates on the late-age concrete strength.

In this study, considering instabilities such as the decrease in the late-age strength of concrete owing to the addition of calcium nitrite, we examined the concrete strength behavior from the viewpoint of hydrate generation. We observed the formation behavior of hydrates from an early age to a late age under low-temperature curing conditions upon adding calcium nitrite as a nitrite-based frost-resistant accelerator. Thermogravimetric-differential thermal analyses and differential thermal gravimetry (TG/DTG), X-ray diffraction (XRD), and solid-state nuclear magnetic resonance (NMR) were used to quantitatively evaluate the hydration products. In addition, the relationship between hydrate formation and the development of the compressive strength characteristic was analyzed through the measurement of compressive strength.

## 2. Experimental

### 2.1. Materials and Procedures

For our experiments, all specimens were prepared at a water/cement ratio of W/C = 0.5 using white Portland cement (wPC, density: 3.07 g/cm^3^, blaine value: 3830 cm^2^/g). Table 1 lists the relevant experimental parameters. Calcium nitrite and small amount of calcium nitrate (CN, Ca(NO_2_)_2_ about 30 wt% and Ca(NO_3_)_2_ about 3 wt% aqueous solution, Nissan chemical corporation, Tokyo, Japan) were used as the nitrite-based frost-resistant accelerator. Table 2 lists the properties of CN used in this study. In order to confirm the change in the hydrate formed as a function of the amount of CN added, the specimens were prepared with different amounts of CN (0 wt%, 4 wt%, 8 wt%; wt% to cement weight) [7]. All specimens were cured under sealed conditions at 10 °C [3]. After curing, the specimens were finely ground to powder, immersed in acetone, and filtered with the use of a Buchner funnel. The chemical experiments were conducted over various curing ages (1 h to 56 d). In this study, “early age” was defined as “within 24 h after mixing”. The compressive strength of mortar was measured, and the relationship between the hydrate formation behavior and strength development was studied. The compressive strength was measured at various curing ages (1 d to 56 d). The fine-aggregate (density: 2.68 g/cm^3^, water absorption ratio: 2.17 wt%)-to-cement ratio of mortar was set to 2.5:1, and the size of each mortar cylinder specimen was diameter 5 cm × height 10 cm.

### 2.2. Compressive Strength

The compressive strength of the mortar was measured (Industrial Series DX600, Instron Japan, Kawasaki, Japan) in accordance with JIS-A-1108 [12] at each age (1, 3, 7, 14, 28, and 56 days). The load was uniformly applied to such a degree that no impact was applied. The loading speed was set to 0.6 ± 0.4 N/mm^2^ per second. The compressive strength was measured five times per each sample level, and the results of compressive strength are represented by three average values excluding minimum and maximum values.

### 2.3. Thermogravimetric/Differential Thermal Gravimetry

TG/DTG (STA 7200, Hitachi, Tokyo, Japan) was performed on samples to examine the thermal decomposition, in a nitrogen atmosphere, from 20 to 1000 °C at a heating rate of 20 °C/min. All measurements were performed with 10 mg of powder; the quantitative of Ca(OH)_2_ in a sample was calculated from weight loss measured from the TG curve around 400 to 480 °C.

### 2.4. X-Ray Diffraction

XRD was performed to identify the changes of crystalline phase. A Rigaku-SmartLab powder diffractometer (Tokyo, Japan) was used for measurements. The XRD conditions were as follows: Cu-Kα radiation resource; 40 kV; 30 mA; scan range, 3 to 70°/2θ; scan speed, 2°/min; step width, 0.02°/step.

### 2.5. Solid-State Nuclear Magnetic Resonance

^27^Al NMR spectra were collected at 208.6 MHz on JEOL ECA-800 (magnetic field 18.8T, Tokyo, Japan) using a 3.2 mmφ probe. The 27Al NMR experiments employed a spinning speed at 20 kHz, a pulse width of 0.9 μs, a relaxation delay of 0.5 s, and a total of 1280 scans. ^29^Si NMR spectra were collected at 99.4 MHz on JEOL ECA-500 (magnetic field 11.75T, Tokyo, Japan) using a 3.2 mmφ probe. The 29Si NMR experiments employed a spinning speed at 10 kHz, a pulse width of 3.6 μs, a relaxation delay of 15 s and a total of 2500 scans. Analysis of the solid-state NMR spectra were performed on a JEOL Delta NMR processing and control software (Delta 5.3.1).

## 3. Results and Analysis

### 3.1. Compressive Strength

**Figure 1:**
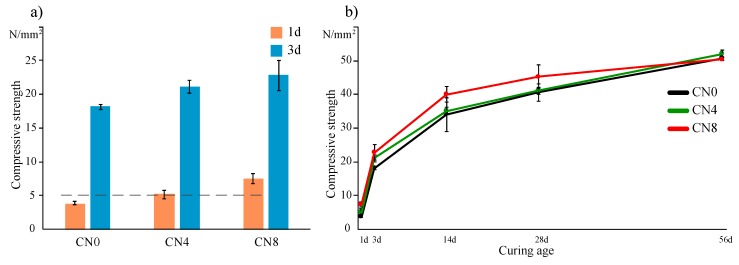
Mortar specimen compressive strength (**a**) on days 1 and 3, and (**b**) at all ages as a function of calcium nitrite amount.

Figure 1a,b show the compressive strength results of the mortar specimens cured at 10 °C for each amount of CN added. As per the Japanese standard, the early (1 d) compressive strength for preventing early frost damage is set to more than 5 N/mm^2^ [3]. The 1-d compressive strength results in the experiment were recorded as 3.8 N/mm^2^ for CN0, 5.2 N/mm^2^ for CN4, and 7.6 N/mm^2^ for CN8, indicating a proportional correlation between the CN amount and the early compressive strength. This proportional tendency appears up to 28 d (CN0: 40.6 N/mm^2^, CN4: 41.1 N/mm^2^, CN8: 45.2 N/mm^2^), and a subsequent reversal in the compressive strength with increasing CN addition occurs at 56 d (CN0: 50.7 N/mm^2^, CN4: 51.9 N/mm^2^, CN8: 50.3 N/mm^2^), but the final difference in strength between the samples is not significant. Under the 10 °C curing condition, the increase in the early strength with increase in the CN amount can be attributed to the increase in hydration products owing to the promotion of the reactions of C_3_A, C_3_S, and *β*C_2_S, as is known from previous studies [9,11,13]. In this study, we further analyze this relationship between hydrate formation and compressive strength development with the aid of experimental results. In addition, we note here that, under the experimental conditions (amount of CN and curing temperature), there was no significant decrease in the compressive strength at a late age (28 d, 56 d).

### 3.2. Hydrate Formation Behavior

#### 3.2.1. TG/DTG

**Figure 2:**
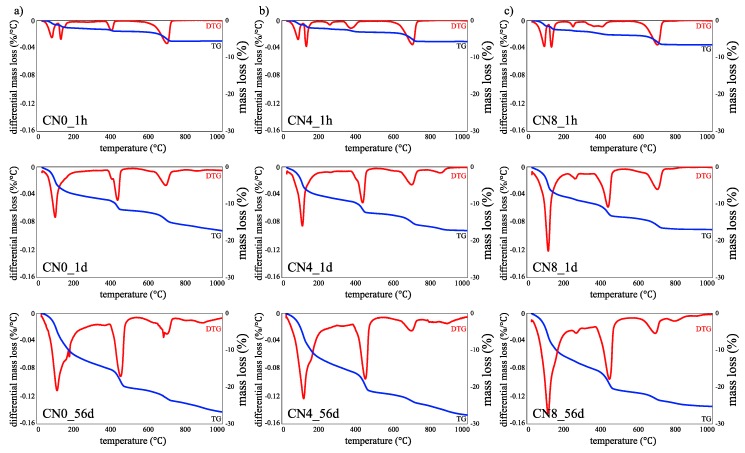
Thermogravimetric/differential thermal gravimetric (TG/DTG) analysis results for specimens at curing ages of (**a**) 1 h, (**b**) 1 d, (**c**) 56 d.

Figure 2 shows an example of the TG/DTG graph as a function of the amount of CN added at curing ages of 1 h, 1 d, and 56 d. We note that the first significant difference in the results as a function of CN addition lies in the decomposition between 200 and 300 °C. In the case of the specimens to which CN was added, a peak of around 260 °C is observed for the curing age of 1 h. As per the literature, this peak can be attributed to synthetic nitrite-AFm, which is formed by the decomposition of hydroxyl and nitrite groups [11,14]. In the case of CN4, the peak is observed for the curing age of 1 d, but not for 3 d. However, in case of CN8, the peak is observed at all ages, and it shows a tendency to increase with increase in the curing age. In addition, the decomposition peak of AFt and AFm near 100 °C can also be confirmed to increase with increase in the CN amount for 1 h, and the difference becomes larger at 1 d [15]. With regard to the 1 d and 56 d results at ~100 °C, we speculate that C-S-H decomposition is also involved [15]. The results of the decomposition of Ca(OH)_2_ in the temperature range of 400–480 °C show a peak at a relatively low temperature (370–420 °C) for the specimens in the aging range of 1 h to 12 h, and a peak at ~390–450 °C for the specimens over the curing ages of 1 d to 56 d. Based on the quantitative evaluation of Ca(OH)_2_, we can observe a slight correlation between the amount of CN addition and Ca(OH)_2_ production for the curing age of 6 h to 12 h (6 h; CN0: 2.7 wt%, CN4: 3.9 wt%, CN8: 6.3 wt%, 12 h; CN0: 5.3 wt%, CN4: 6.2 wt%, CN8: 9.3 wt%), but the difference in Ca(OH)_2_ production gradually decreases over the curing age of 1 d to 56 d (1 d; CN0: 10.9 wt%, CN4: 13.7 wt%, CN8: 13.7 wt%, 56 d; CN0: 20.4 wt%, CN4: 22.0 wt%, CN8: 21.9 wt%). Based on these results, we posit that CN addition not only promotes the reaction of C_3_A but also the initial generation (6 h to 12 h) of Ca(OH)_2_; however, there is no significant difference in the Ca(OH)_2_ amount produced for all ages [9,11,13].

In the range of 20–1000 °C, each specimen shows similar water loss for all ages (1 h: ~6 wt%, 1 d: ~17 wt%, 56 d: ~ 25 wt%). This result is thought to negligibly affect the total bonding water at later ages, even if the production of nitrite-AFm and AFt increases in the early age with CN addition. However, this result indicates that the amount of binding of C-S-H gel and other hydrates may be relatively reduced under the influence of the large amounts of nitrite-AFm and AFt generated [8,11,13,16].

#### 3.2.2. XRD

**Figure 3:**
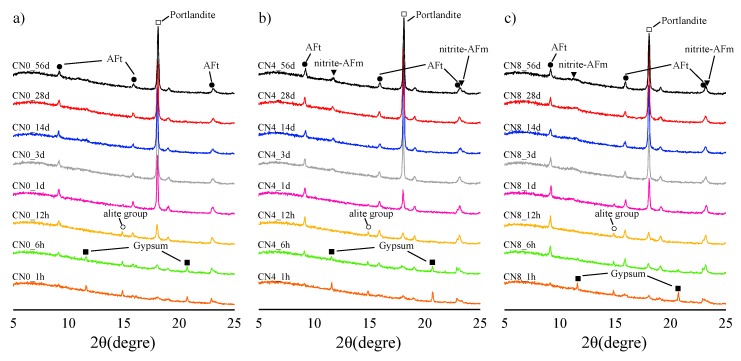
X-ray diffraction (XRD) results for (**a**) CN0, (**b**) CN4, and (**c**) CN8 samples.

Figure 3 shows the XRD results in the diffraction angle range of 5–25° for each curing age. At the curing age of 1 h, AFt (2θ deg 9.2°, 16.1°, 23.2°) and gypsum (2θ deg 11.6°, 20.7°) peaks are visible for all specimens. In the case of the CN4 and CN8 specimens, nitrite-AFm (2θ deg 11.0° to 11.2°, 23.5° to 23.7°) peaks are also visible along with AFt [16,17,18]. In this regard, Balonis confirmed the generation of AFt and nitrite-AFm via the ion exchange reactions of SO_4_^−^and 2NO_2_^−^ of AFm through the reaction experiment involving synthetic AFm and calcium nitrite [11,14]. In our case, we speculate that the C_3_A reaction is accelerated by the addition of CN, and the generation of AFt and nitrite-AFm proceeds simultaneously. In addition, although quantitative evaluation was difficult, weak peaks of gypsum were observed in the CN0 and CN4 specimens at the curing age of 6 h, but not for CN8. In the range of the addition amount of CN8, it is expected that the consumption of gypsum is complete within 6 h via promotion of the reaction with C_3_A. In fact, no gypsum peak was detected after 12 h in all samples. In the hydration process corresponding to 1 h–6 h, the reaction of C_3_A with gypsum and the formation of nitrite-AFm by the addition of CN are expected to significantly affect the early strength enhancement. Under the curing conditions of this study, beyond the curing age of 1 d, a tendency to decrease slightly with AFt and slightly increase with nitrite-AFm with an increase in the curing age was observed; our quantitative evaluation is discussed later in the paper with regard to the ^27^Al NMR results. Additionally, there was no correlation between the amount of Ca(OH)_2_ (2θ deg 18.0° b) produced and the amount of CN added. This result shows a slightly different tendency from the TG/DTG results, and it is thought that there may be a difference in the crystallinity of Ca(OH)_2_ at an early age owing to the addition of CN; further investigations are necessary in this direction.

#### 3.2.3. Al MAS NMR

**Figure 4:**
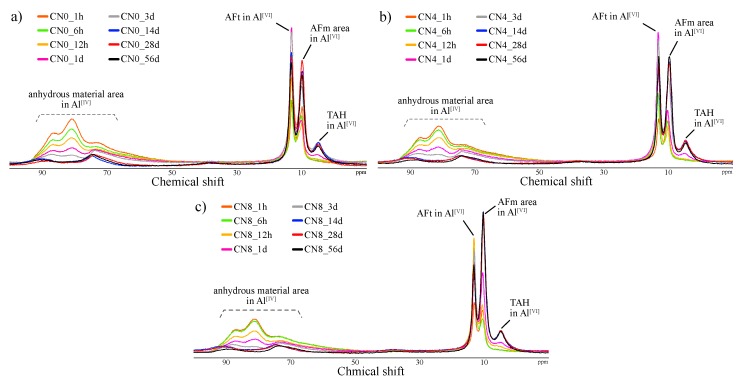
^27^Al nuclear magnetic resonance (NMR) spectra at all ages for (**a**) CN0, (**b**) CN4, and (**c**) CN8 samples.

**Figure 5:**
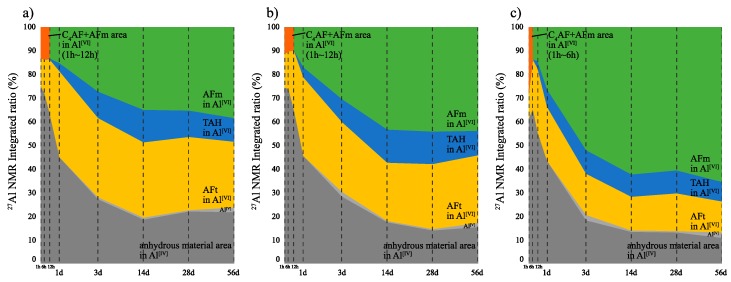
^27^Al nuclear magnetic resonance (NMR) integrated area ratio of (**a**) CN0, (**b**) CN4, and (**c**) CN8 samples.

**Figure 6:**
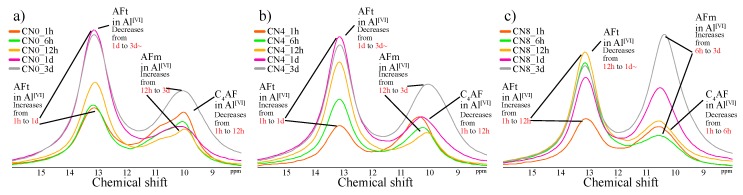
^27^Al nuclear magnetic resonance (NMR) spectra of Al^[VI]^ in range from 1 h to 3 d for (**a**) CN0, (**b**) CN4, and (**c**) CN8 samples.

**Figure 7:**
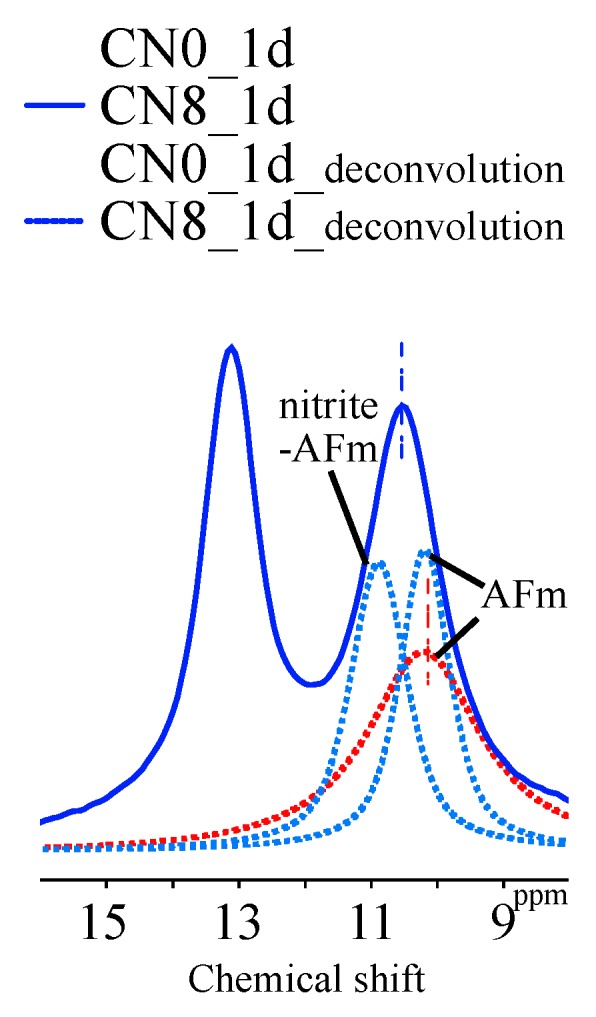
^27^Al nuclear magnetic resonance (NMR) spectra of CN0 and CN8 specimens after 1 d (8 ppm to 16 ppm).

Figure 4 and Figure 5 show the solid-state ^27^Al NMR spectra and integrated area ratio corresponding to Al resonance, respectively. The chemical shift regions for ^27^Al NMR can be described as follows: tetrahedral coordination (Al^[IV]^), 50 to 100 ppm; pentahedral coordination (Al^[V]^), 30 to 40 ppm; octahedral coordination (Al^[VI]^), 10 to 20 ppm [19,20]. In general, the broad range of resonance from 50 to 100 ppm can be attributed to structures with reduced crystallinity including Al in the anhydrous material [21,22]. In this study, Al^[IV]^ resonance at an early age corresponds to anhydrous materials. The resonances at 9.7 ppm, corresponding to monosulfate, and 12.4 ppm, corresponding to ettringite, are confirmed in the Al^[VI]^ range [19,20], and the resonance at 5 ppm corresponds to a third aluminate hydrate (Al(OH)_6_^3−^, O_x_Al(OH)_6−x_^(3+x)−^, TAH) [22,23]. Furthermore, the 9 ppm resonance in the Al^[VI]^ regime at an early age can be attributed to C_4_AF (Figure 6). Overall, the CN8 results show a strong tendency toward AFm-related resonance in the Al^[VI]^ range to emerge from the early ages. In the integrated resonance ratio range corresponding to 1 h to 6 h, the Al^[IV]^ resonance is 75 wt% for CN0, 74 wt% for CN4, and 61 wt% for CN8, that is, CN8 addition accelerates the hydration of Al-based anhydrous material. However, at the curing age of 1 d, the ratio of Al^[IV]^ resonance is similar between all the samples, with 45 wt% for CN0, 45 wt% for CN4, and 43 wt% for CN8. Furthermore, the consumption of Al^[VI]^ in C_4_AF at an early age tends to extend up to 12 h for CN0 and CN4, but this consumption is terminated at 6 h for CN8 (Figure 6). This result shows a tendency similar to the consumption period of gypsum identified as per the XRD results, and it is expected that the reaction of C_3_A, C_4_AF, and gypsum will be accelerated up to 6 h in the range of CN8 addition, thus affecting the early strength enhancement. Figure 6 shows the solid-state ^27^Al NMR spectra of the Al^[VI]^ area for the range of 1 h to 3 d. The biggest difference regarding the presence or absence of CN is the proportion of the AFt and AFm resonances. In the case of CN8, the area of AFm is wider than that of AFt in comparison with CN0 and CN4. With respect to the AFm resonance, the center of the resonance moves toward a higher ppm value in the case of CN8 when compared with the corresponding resonance center for CN0, as shown in Figure 7. This result is due to an overlap between AFm (9.7–10.3 ppm) and a different bonding structure detected in the range of 10.7–10.9 ppm, which can be attributed to nitrite-AFm, thereby resulting in a broader resonance. However, it was difficult to separate the resonances under the present experimental conditions, and the resonance over 9.7 to 10.9 ppm was expressed as AFm(Total). The 1 d results showed that the AFt and AFm proportions were AFt: 36 wt%, AFm: 15 wt% for CN0, AFt: 33 wt%, AFm(Total): 16 wt% for CN4, and AFt: 23 wt%, AFm(Total): 26 wt% for CN8. In the case of CN0 and CN4, the AFt resonance increases from 1 h to 1 d and then tends to decrease slightly. AFm resonance develops from 12 h to 3 d. On the other hand, in the case of CN8, the development of AFt resonance runs from 1 h to 12 h and subsequently decreases. Furthermore, in CN8, AFm resonance is observed to develop steeply from 6 h onward (Figure 6).

From these results, we speculate that the difference in compressive strength at 1 d significantly affects the proportion of AFt and AFm(Total), rather than the increase in the amount of total hydrates produced by the hydration promotion of C_3_A. This production proportion is maintained until the curing age of 56 d. The relative increase in AFm(Total) contributes to the development of compressive strength in early aging when C-S-H production is not sufficient, and this effect decreases with increasing C-S-H as the curing age progresses.

#### 3.2.4. Si MAS NMR

**Figure 8:**
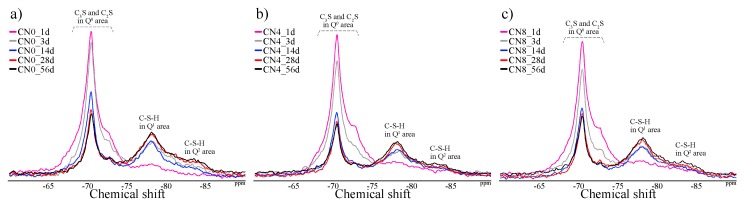
^29^Si nuclear magnetic resonance (NMR) spectra of (**a**) CN0, (**b**) CN4, and (**c**) CN8 samples.

**Figure 9:**
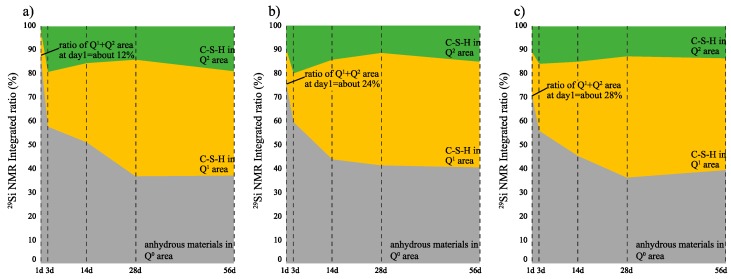
^29^Si nuclear magnetic resonance (NMR) integrated area ratio of (**a**) CN0, (**b**) CN4, and (**c**) CN8 samples.

Figure 8 and Figure 9 show the solid-state ^29^Si NMR spectra and integrated area ratio of Si resonance, respectively. We note that Q^0^ and Q^1^–Q^2^ resonances are detected at around −70 to −73 ppm and −79 to −85 ppm, respectively [24,25]. As per the ^29^Si NMR results for the curing age of 6 h, Q_1_ and Q_2_ resonances are not detected in all samples. However, Q^1^ and Q_2_ resonances are detected at age 1 d in all samples, and the combined ratio of Q^1^ + Q^2^ is 12 wt% in CN0, 24 wt% in CN4, and 28 wt% in CN8, which is proportional to the amount of CN added. As with the trend of the ^27^Al NMR spectra results, this proportional tendency gradually reduces after 1 d. The 3 d to 28 d results showed that the combined ratio of Q^1^ and Q^2^ increases gradually but shows similar binding rates in all samples (Figure 9, 3 d: ~43 wt%, 14 d: 48 wt%, 28 d: 62 wt%). In addition, the ratio tends to slightly reverse at the curing age of 56 d, but the binding rate difference is not significant. Upon comparison of the compressive strength corresponding to the ^27^Al NMR and ^29^Si NMR integrated area ratios, it is observed that the improvement in compressive strength at an early age (1 d) can be explained only by the relative increase in C-S-H with CN addition. However, in regard to the 3 d compressive strength results, although the amount of C-S-H production is similar among all three samples, the compressive strength increases with the addition of CN. From this result, we can confirm that the formation of AFm(Total) also affects the early compressive strength up to 1–3 d. Additionally, although the difference in compressive strength is not large, the results show that the compressive strength and the amount of C-S-H produced over 28 d to 56 d are reversed. Consequently, the addition of a large amount of CN may interfere with the production of C-S-H at a late age owing to the large amount of AFm(Total) produced at an early age. In this regard, as per Choi [16], the compressive strength is significantly decreased in the late aging of the specimens containing 13 wt% CN additive or more. However, in our case, there was no noticeable decrease in compressive strength and no decrease in the C-S-H production over the range of CN addition considered in the study.

## 4. Conclusions

In this study, we performed various types of analysis to understand the relationship between the hydrate formation behavior and strength development in the early age range of concrete as a function of the quantity of frost-resistant accelerator, calcium nitrite, added. The results of the study are summarized as follows:

(1) In the compressive strength experiments on the mortar specimens, the early compressive strength was proportional to the amount of CN added, and this addition was found to be effective in preventing early freezing damage. However, this correlation gradually decreases with curing age.

(2) From the TG/DTG results, we confirmed the decomposition of the nitrite-AFm group from the age of 1 h for CN-added samples. Furthermore, this decomposition is expected to affect early compressive strength development. It was observed that the total amount of binding water was similar regardless of the amount of CN addition for all curing ages, indicating the possibility of a cyclic increase and decrease in the binding water between each hydrate by the amount of CN addition.

(3) From the XRD results, we confirmed that the consumption of gypsum is accelerated and nitrite-AFm is produced at the age of 1 h upon CN addition. However, no significant compressive strength development factor was found in the formation of Ca(OH)_2_.

(4) While the ^27^Al NMR results showed that the C_3_A reaction was accelerated upon CN addition over the range of 1–6 h, all samples showed a similar hydration progress at the age of 1 d. However, the relative development ratio of AFm to AFt rapidly increased upon CN addition, possibly strongly affecting the early compressive strength.

(5) As per the ^29^Si NMR results, we found that the production of C-S-H is accelerated by CN addition at an early age (Day 1), thereby contributing to the early compressive strength development. However, this advantage gradually decreased with increase in age, and the trend appeared to slightly reverse with further increase in the age. This reversal is most likely related to the decrease in compressive strength at a late age by the addition of large amounts of CN [16].

## Figures and Tables

**Table 1:** Specimen parameters utilized in experimental design.

Type	Index	CN Content [Cement × wt%]	W/C wt%	Curing Condition	Curing Age h: hour, d: day		Analysis Method
Cement paste	CN0 CN4 CN8	0 4 8	50	+10 °C Sealed	1 h 6 h 12 h	1 d 3 d 14 d 28 d 56 d	TG/DTG XRD ^27^Al MAS NMR ^29^Si MAS NMR
Mortar					-	1 d 3 d 14 d 28 d 56 d	Compressive Strength

Note: CN: nitrite-based frost-resistant accelerator (aqueous solution); CN0: Mixing amount of CN = 0%; CN4: Mixing amount of CN = 4%; CN8: Mixing amount of CN = 8%.

**Table 2:** Properties of the nitrite-based frost-resistant accelerator.

Component	Component Ratio	Specific Gravity of Aqueous Solution	pH of Aqueous Solution
Ca(NO_2_)_2_	31.84 wt%	1.308	10.5
Ca(NO_3_)_2_	3.17 wt%		
